# Dynamic Oscillatory Signatures of Central Neuropathic Pain in Spinal Cord Injury

**DOI:** 10.1016/j.jpain.2014.02.005

**Published:** 2014-06

**Authors:** Aleksandra Vuckovic, Muhammad A. Hasan, Matthew Fraser, Bernard A. Conway, Bahman Nasseroleslami, David B. Allan

**Affiliations:** ∗ Biomedical Engineering Division, University of Glasgow, Glasgow, United Kingdom; † Queen Elizabeth National Spinal Injuries Unit, Southern General Hospital, Glasgow, United Kingdom; ‡ Department of Biomedical Engineering, University of Strathclyde, Glasgow, United Kingdom; § Department of Biomedical Engineering, NED University of Engineering and Technology, Karachi, Pakistan; ‖ Department of Biology, Northeastern University, Boston, Massachusetts

**Keywords:** Central neuropathic pain, spinal cord injury, event-related synchronization/desynchronization, motor imagery, electroencephalography

## Abstract

Central neuropathic pain (CNP) is believed to be accompanied by increased activation of the sensorimotor cortex. Our knowledge of this interaction is based mainly on functional magnetic resonance imaging studies, but there is little direct evidence on how these changes manifest in terms of dynamic neuronal activity. This study reports on the presence of transient electroencephalography (EEG)-based measures of brain activity during motor imagery in spinal cord–injured patients with CNP. We analyzed dynamic EEG responses during imaginary movements of arms and legs in 3 groups of 10 volunteers each, comprising able-bodied people, paraplegic patients with CNP (lower abdomen and legs), and paraplegic patients without CNP. Paraplegic patients with CNP had increased event-related desynchronization in the theta, alpha, and beta bands (16–24 Hz) during imagination of movement of both nonpainful (arms) and painful limbs (legs). Compared to patients with CNP, paraplegics with no pain showed a much reduced power in relaxed state and reduced event-related desynchronization during imagination of movement. Understanding these complex dynamic, frequency-specific activations in CNP in the absence of nociceptive stimuli could inform the design of interventional therapies for patients with CNP and possibly further understanding of the mechanisms involved.

**Perspective:**

This study compares the EEG activity of spinal cord–injured patients with CNP to that of spinal cord–injured patients with no pain and also to that of able-bodied people. The study shows that the presence of CNP itself leads to frequency-specific EEG signatures that could be used to monitor CNP and inform neuromodulatory treatments of this type of pain.

Central neuropathic pain (CNP) is caused by an injury to the somatosensory system^3^, ^19^ and has a high prevalence in patients suffering from amputation (80%),^15^ spinal cord injury (SCI; 40%),^47^ multiple sclerosis (27%),^43^ Parkinson disease (10%),^6^ and stroke (8%).^2^ Its symptoms do not respond well to medication, and the drugs used are often associated with significant adverse effects.^4^, ^37^, ^57^ This has generated interest in non–drug-based treatment methods such as cognitive-behavioral therapies^21^, ^23^ and interventions such as repetitive transcranial magnetic stimulation (rTMS),^20^, ^21^, ^29^, ^30^, ^38^, ^45^, ^52^ transcranial direct current stimulation (tDCS),^10^, ^12^, ^21^, ^29^, ^38^, ^45^ and neurofeedback (NF).^22^, ^25^, ^27^, ^48^, ^56^ Although multiple studies have confirmed efficiency of these stimulation interventions for various types of acute or chronic pain, including CNP,^10^, ^20^, ^25^, ^26^, ^30^, ^45^, ^56^ the stimulation parameters and spatial targets are often determined heuristically.^25^, ^29^, ^30^

Many studies have shown a correlation between CNP and reorganization of the sensorimotor cortex^15^, ^17^, ^59^ where, because of sensory loss caused by the injury, the affected cortical somatotopy undergoes remapping or reorganization.^59^ Comparative functional magnetic resonance imaging (fMRI) studies involving SCI patients demonstrate that during the performance of imagined movements, those patients with CNP show activation of brain areas related to both motor imagery (MI) and pain processing.^18^ It has now been proposed that the intensity of the perceived pain is proportional to the extent of reorganization influencing cortical sensorimotor processing.^59^ However, the observation that phantom pain in amputees correlates more strongly with maintained phantom representation than with a remapped representation of intact body parts contradicts this hypothesis.^32^ Nevertheless, the above results indicate that relationships exist between the pathology underlying CNP and long-term adaptive changes in cortical activation associated with sensorimotor behavior even in the absence of a painful peripheral stimulation.

Although fMRI studies of patients with CNP can provide an anatomic spatial focus for further investigations, the method cannot provide the temporal resolution needed to understand the dynamics of the activation patterns that may exist in CNP. In this regard, electroencephalography (EEG) can provide a useful noninvasive basis for experimental investigation. On the other hand, EEG has a very limited spatial resolution as it records the electrical activity from the surface of the skull, thus measuring the combined activity of near and distant cortical sources. Furthermore, EEG measures only surface cortical activity, and as a result the activity of deeper cortical structures involved in processing of chronic pain, such as anterior cingulate cortex and insular cortex, cannot be measured.

At present, EEG recordings of patients with CNP have been limited to studies of resting EEG in eyes open (EO) and eyes closed (EC) states,^11^, ^24^, ^36^, ^46^ suggesting that the increased power in the theta range and decreased frequency of the dominant alpha rhythm are major signatures of CNP. These observed changes in EEG power were widespread and not restricted to any specific area of the cortex.^11^, ^24^, ^36^, ^46^

Although these studies demonstrate altered EEG activity in resting states, they do not attempt to explore how CNP influences brain activation patterns during performance of tasks that require sensorimotor processing analogous to those used in fMRI studies.^18^, ^59^ Accordingly, we undertook this EEG-based study to quantitatively examine the brain activation patterns associated with the presence and absence of CNP in patients with SCI while they performed imagined motor tasks.

MI induces dynamic activation of sensorimotor cortical areas that can be recorded by EEG in healthy subjects and in patients with paralysis due to SCI. The use of MI as an activation probe and EEG as the recording modality therefore presents a simple noninvasive way to explore the comparative cortical activation patterns that accompany MI in patients with and without CNP. This study's principal aim was to examine the evidence for altered cortical activations in patients with and without CNP in the absence of peripheral nociceptive stimulation. Our final goal was to determine if EEG-based electrophysiological markers of the condition can be identified and whether this knowledge can assist in designing more effective rTMS, tDCS, or NF treatment interventions for CNP.

## Methods

### Participants

A total of 30 age-matched adult (between 18 and 55 years old) volunteers were recruited in 3 groups of 10. The groups were as follows:

1. Paraplegic patients with diagnosed CNP below the level of their spinal cord injury (3 female [F], 7 male [M], age 45.2 ± 9.1 [mean ± standard deviation])
2. Paraplegic patients with no chronic pain (2 F, 8 M, age 44.4 ± 8.1)
3. Able-bodied volunteers with no chronic pain (3 F, 7 M, age 39.1 ± 10.1)

The neurologic level of SCI was determined using the American Spinal Injury Association (ASIA) Impairment Classification.^33^ All SCI patients were at least 1 year postinjury and had a spinal lesion at or below T1. Inclusion criteria for patients with CNP were a positive diagnosis of CNP; a reported pain level ≥5 on the visual numerical scale; and a treatment history of CNP for at least 6 months. The general exclusion criteria for all 3 groups were a presence of any chronic (non-CNP) or acute pain at the time of the experiment; brain injury; or other known neurology that would affect EEG interpretation or would prevent patients from understanding the experimental task. Information on both patient groups is shown in Tables 1 and 2.

**Table 1:** Information About Patients With CNP (PWP Group)

No.	Level of Injury	ASIA Classification	Years After Injury	Pain VNS	Years With Pain	Medications
1	T5	A	7	7	7	Baclofen, carbamazepine, gabapentin
2	T5/6	A	11	6	11	None
3	T5	A	7	8	7	Pregabalin, gabapentin
4	L1	B	15	7	15	Gabapentin
5	T6/T7	D	4	7	3	Pregabalin
6	T7	B	6	8	5	None
7	T6/7	B	25	10	24	Gabapentin
8	T1	A	25	5	10	Pregabalin
9	T5	A	14	5	13	Amitriptyline, baclofen, diazepam
10	L1	B	5	5	4	None

Abbreviation: VNS, visual numerical scale.

**Table 2:** Information About Patients With No Pain (PNP Group)

No.	Levelof Injury	ASIA Classification	Years After Injury
1	T7	A	7
2	T7	B	7
3	T12	A	7
4	L1	A	6
5	T2	A	2
6	T5	B	15
7	T11	A	11
8	T4	A	9
9	T7	A	15
10	T7	B	22

Informed consent was obtained from all participants, and ethical approval was obtained from the university ethical committee for the able-bodied group and from the National Health Service ethical committee for the patient groups.

### Recording Equipment

A 61-channel EEG (Synamp 2; NeuroScan, Charlotte, NC) was recorded with electrodes placed according to standard 10-10 locations^1^ using an ear-linked reference and AFz ground. Electro-oculogram was recorded from 3 channels around the right eye. All channels were sampled at 1,000 Hz. Individual electrode impedance was below 5kΩ. In addition, electromyograms were recorded from the right and the left wrist extensor muscles and right shank using the bipolar inputs to the Synamp device. The purpose of electromyography recording was to check for the absence of any evidence of voluntary movements when subjects attempted MI.

### Experimental Study Design

Participants were instructed not to drink coffee or alcohol on the day of the experiment. EEG was recorded in 2 paradigms: spontaneous activity and induced activity during cue-based MI. Before starting the experiment, participants with pain were asked to fill out a Brief Pain Questionnaire^13^ to establish the level and location of pain.

### Spontaneous EEG Recording

Spontaneous resting EEG was recorded under the EO and EC conditions in a quiet room. During the EO state, participants were asked to visually fixate on a small cross presented on a computer screen, whereas in the EC state they had to close their eyes and relax. EEG was recorded for 2 minutes for each condition repeated 3 times, alternating between the conditions.

### Cue-Based MI

**Figure 1:**
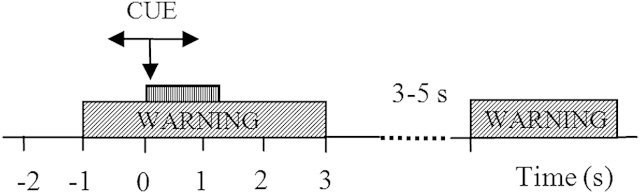
Experimental setup: At t = −1 second, a readiness cue (a cross) appeared on a computer screen, followed by a cue (an arrow) at t = 0 second. The cue stayed on the screen until t = 1.25 seconds, whereas the warning stayed until t = 3 seconds. A volunteer was asked to perform repetitive imagination of movement from t = 0 second until the readiness cue disappeared at t = 3 seconds. Different arrows indicate motor imagery of different limbs.

An experimental protocol that instructed participants to imagine hand or lower limb movements was devised using visual cues. Participants were seated at a desk, approximately 1.5 m in front of a computer monitor. Participants were instructed to look at the center of the monitor and were instructed to respond to a sequence of visual cues. The cues included at t = −1 second a readiness cue (a cross), which remained on for 4 seconds (Fig 1). At t = 0 second an initiation cue, presented as an arrow, was displayed for 1.25 seconds, pointing to the left ←, to the right → or down ↓ and corresponding to imagination of the left hand waving, right hand waving, and tapping with both feet. Participants were asked to continue to perform imaginary movements until the cross disappeared from the screen (3 seconds after the initiation cue appeared).

In total, 60 trials of each movement type were presented to subjects, and cues were collected in randomized sequences comprising 10 trials with rest periods between.

### Data Pre-Processing

For pre-processing of spontaneous EEG, a high pass filter (IIR, 12db cutoff frequency) was set to 1 Hz and a notch filter was applied between 48 and 52 Hz to remove line noise at 50 Hz. Filtering was applied forward and then backward to avoid phase shift. Signals were then down-sampled to 250 Hz. EEG was visually inspected, and sequences containing electro-oculogram artifact and other types of noise (amplitude exceeding approximately 100 μV over all channels) were manually removed. For EO and EC states, for each volunteer after noise removal, a minimum of 3 minutes of data was required for data inclusion. For pre-processing EEG data during MI, signals were pre-processed as explained above and were then exported to EEGLab.^14^ Independent component analysis was performed using the Infomax algorithm^7^ implemented in EEGLab for advanced noise removing purposes to avoid excessive EEG removal from a limited number of trials. In this way no more than 2 (of 60) trials had to be removed per data set.

### Analysis of Spontaneous EEG

Data were re-referenced to an average reference. For each volunteer, a power spectral density (PSD) was calculated over 2-second windows overlapped for 1 second using Hamming windows (MathWorks, Natick, MA). Logarithmic PSD was calculated as 10∙log_10_PSD for normalization purposes. The location of the dominant alpha peak was determined based on PSD. Location of a peak frequency was additionally confirmed by a visual inspection. A dominant peak frequency for each volunteer was normalized and averaged over all electrodes.

A “study” structure was created in EEGLab to compare on a group level between different conditions and different groups. “Groups” were 3 groups of volunteers (able-bodied [AB], patients with pain [PWP], and patients with no pain [PNP]), whereas “conditions” were EO and EC states. PSD was averaged over different frequency bands and compared for each electrode location between groups and between conditions. To compare between means of 2 variables, a nonparametric permutation test^9^ based on resampling was implemented in EEGLab with a significance level set to *P* = .05. A nonparametric 2-way analysis of variance based on permutation analysis was also applied to compare between groups and conditions and to check for their interaction. Performing of each possible permutation for 3 groups would be computationally extensive; therefore, the Monte Carlo method was used. A correction for multiple comparisons was performed using the false discovery rate (FDR).^8^ All procedures were implemented in EEGLab.

### EEG Analysis of MI

Before performing the analysis, EEG data were re-referenced to the average reference. A “study” structure was designed in EEGLab to allow EEG analysis on a group level. “Groups” were PWP, PNP, and AB groups, and “conditions” were MI of left hand waving, right hand waving, and tapping with both feet. Data analysis was based on ERD/ERS phenomena,^44^ which we briefly explain here. During MI of a limb, neuronal firing is desynchronized, resulting in a reduced amplitude and energy of the measured EEG signal in the sensorimotor rhythms (8–12 Hz and 16–24 Hz) as compared to the energy level in the reference period before MI. This phenomenon is called event-related desynchronization (ERD). It should be noted that suppression of energy actually corresponds to the active, not the idle, brain state. The opposite phenomenon, increased synchrony resulting in increased energy level, is called event-related synchronization (ERS) and is often observed in the cortical areas surrounding the areas under desynchronization.^39^ In its simplified version, for a chosen frequency band, ERS/ERD is calculated as

$$ERS/ERD\%=\frac{\left(E-R\right)}{R}$$

where E is “an event”—for example, MI—and R is a “reference period” preceding the event. An extension of the ERS/ERD, called event-related spectral perturbation (ERSP), based on sinusoidal wavelets rather than on filters,^31^ was used to allow more precise time-frequency analysis. For calculating the ERS/ERD of each single volunteer, a reference period from −1.9 to −1.1 seconds (before the cross) was adopted, and time-frequency decomposition was performed in a frequency range 3 to 55 Hz using a sinusoidal wavelet with minimum 3 wavelet cycles per data window at lowest frequencies. Overlapping Hanning tappers windows were applied.

In order to find regions of significant ERS/ERD for each condition (on a single electrode site), a significance level was set to *P* = .05 and nonparametric bootstrapping procedure (N = 2,000 trials)^16^ was performed, comparing ERD/ERS maps between groups. An FDR correction was applied to correct for multiple comparison from multiple time-frequency windows.

Scalp maps were created based on ERS/ERD averaged over certain frequency bands and short time windows (200 ms). Comparison between scalp maps of different groups or conditions was performed based on a permutation statistics (*P* = .05) as previously described, and FDR was applied to account for comparison from multiple electrode sites.

Although it is believed that increased ERD corresponds to active brain state, ERS/ERD scalp maps are only an approximation of the surface cortical activation, limited to electrode locations defined by a 10-10 system.^1^ Additional possible inaccuracy is caused by the fact that a single electrode can record the activity of several sources. Therefore, although in the Results section we present ERS/ERD maps from single electrodes located over the primary motor cortex, we do not assume that they reflect cortical activity restricted to the cortical areas lying directly under the electrodes.

## Results

**Figure 2:**
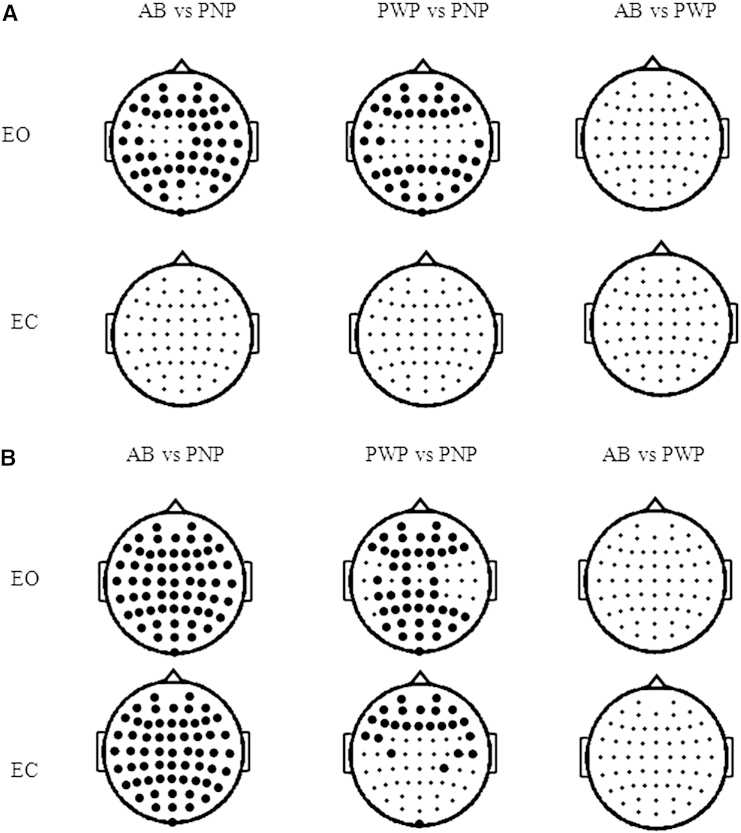
Areas of statistically significant difference between PSD in the EO and EC states between each combination of 2 groups (*P* = .05) with FDR correction for multiple comparison. **(A)** Theta band 4 to 8 Hz. **(B)** Alpha band 8 to 12 Hz. Large black dots mark electrode locations with statistically significant differences between groups.

To assess the dynamic response of the motor cortex to CNP in an imagined movement task, we first characterized the relaxed states, using EO and EC states. The PSD in the theta and alpha bands was compared between groups for each combination of 2 groups (AB vs PNP, AB vs PWP, PWP vs PNP) over all 61 electrode locations (Fig 2). Fig 2A shows differences in the theta band (4–8 Hz) separately in EO and EC states. Fig 2B shows differences in the alpha (8–12 Hz) band in EO and EC states. Black dots show electrode location with statistically significant differences among 2 groups. Because of multiple comparisons across 61 electrodes, an FDR method was used to avoid type II error. These may lead to more conservative results than in previous studies, which have not used FDR. The PNP group had lowest theta and alpha power in the EO state, significantly lower than PWP and AB. Results confirmed that in the EO state, PWP had increased theta PSD compared to PNP group^11^, ^24^, ^36^, ^46^ (Fig 2A, upper row) and comparable theta PSD to the AB group. No difference among groups was found between theta PSDs in the EC state (Fig 2A, lower row). The intensity of the alpha PSD in PWP was comparable with the alpha PSD in the AB group in both EO and EC states (Fig 2B). PWP had larger alpha PSD in the EO state than did the PNP group over most of recording sites (Fig 2B, upper row). However, in the EC state there was no difference between the PWP and PNP groups in the parieto-occipital region (Fig 2B, lower row), which is normally an area of largest alpha activity in the EC state. This effectively means that the PWP group had a reduced EC/EO ratio in the parieto-occipital region. Reduced EC/EO ratio has already been reported in paraplegic patients with CNP and is believed to be an indicator of the thalamocortical network involved in CNP processing.^11^ There was no significant difference between groups in the beta range. In PWP, the dominant frequency 9.1 ± .8 Hz was significantly lower than 10.1 ± .6 Hz in the AB (Wilcoxon *P* = .008) and 10.4 ± .9 Hz in PNP (Wilcoxon *P* = .0085).

### Dynamic Activation of Sensorimotor Cortex During MI

**Figure 3:**
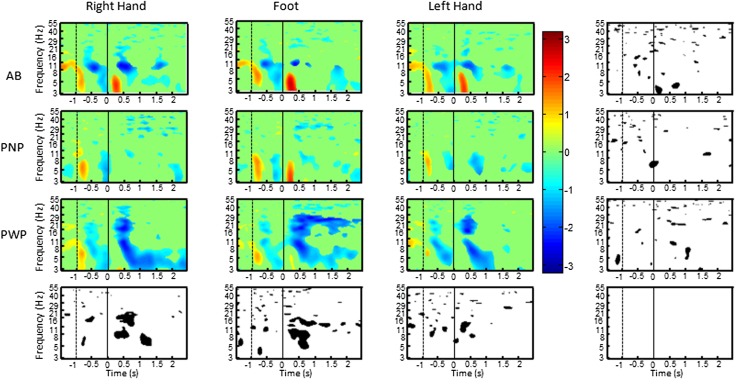
ERS/ERD time frequency map over electrode location Cz, for all 3 groups of participants and for all 3 MI tasks. Figures at the far right show areas of statistically significant differences between the tasks, whereas figures at the bottom row show areas of statistically significant differences among the groups (*P* = .05) with FDR correction for multiple comparison. ERD/ERS map shows a time period starting −2 seconds before the cue and ending 2.5 seconds after the cue in a frequency range 3 to 55 Hz. Participants were asked to start with MI at t = 0 second (dashed line) and to continue with MI until t = 3 seconds.

Fig 3 shows ERS/ERD at electrode location Cz, being of primary somatotopic relevance to the leg area. PWP showed the most significant ERD, spreading over all frequency bands (being most pronounced for the movements of the feet), being statistically significantly larger than ERD in the other groups. This strong ERD persisted during MI of both painful and nonpainful limbs.

### CNP Leads to a Distinctive Cortical Activation

**Figure 4:**
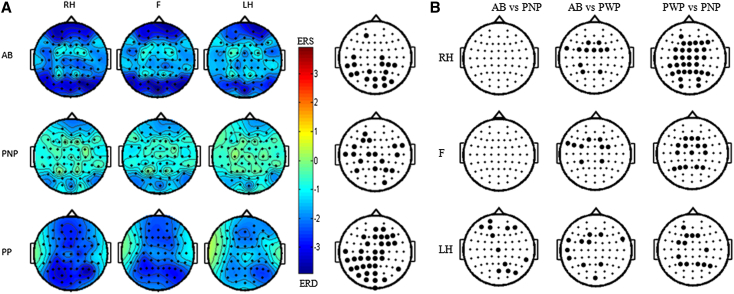
**(A)** Scalp maps of ERS/ERD in 8 to 12 Hz band at 400 to 600 ms post-cue for all 3 groups and all 3 tasks. ERD shows areas of increased cortical activity, as compared to the period before MI. Column at the far right shows areas of statistically significant differences (*P* = .05) with FRD correction for multiple comparison among the 3 tasks. **(B)** Areas of statistically significant difference between groups and tasks are shown in Fig 4A (*P* = .05) with FDR correction for multiple comparison. Abbreviations: RH, right hand; F, feet; LH, left hand.

Cortical activation during imagined movements in patients with CNP was stronger and spatially different from that of the other groups (AB, PNP). Fig 4A shows ERS/ERD scalp maps averaged over the 8 to 12 Hz band and over a period 400 to 600 ms after presentation of a cue on the computer screen, for all groups and all 3 tasks. As this latency period exceeds what would be a normal reaction time to a movement, we believe that this period, 400 to 600 ms after MI cue, corresponds to the covert, that is, “mental” execution of the MI task. This period is also the time point at which intensity of ERD is maximal (Fig 3).

In PWP, ERD was not limited to the cortical presentations of the painful legs. They had a widespread ERD, strongest for MI of the right hand and weakest for MI of the left hand, with no ERS in the surrounding areas (Fig 3A, bottom row). Although shifted posteriorly, the ERD spatial distribution in PWP still follows the somatotopic presentation, where the movement of the right hand causes a strongest ERD at electrode locations placed over the left hemisphere, over the centro-parietal area for the feet, and over the right hemisphere for the left hand. In contrast, AB participants and paralyzed PNP exhibited similar spatial distributions—central ERD accompanied by weak ERS in the areas surrounding the central area (a phenomenon known as “central ERD with surrounding ERS”).^39^ In contrast to relaxed state, where AB and PNP alpha PSD showed statistically significant difference over all cortical areas (Fig 2A), during MI, AB had stronger responses than PNP for MI of the left hand only (Fig 4). The largest difference between PWP and PNP was found for MI of the right hand, although areas of statistically significant difference existed for MI of both hands and of the feet (Fig 4B column PWP vs PNP). Although there was no statistically significant difference in the normalized alpha power in the EO state between AB and PWP (Fig 2), there was a statistically significant difference in the alpha ERD during MI (Fig 4). Statistically significant differences between AB participants and PWP were found for MI of the feet and of both hands—again indicating that CNP produces a widespread increased activity in the sensorimotor cortex. In the AB group, the strongest ERD could be noticed in the frontal and occipital areas. This might be attributed to visual processing of the target and movement planning. A similar but weaker tendency can be noticed in the PNP group. In the PWP group, however, ERD can be noticed over almost all cortical regions from which EEG was measured. A relatively conservative correction for multiple comparisons, which does not take into account spatial correlation of measured values, might explain why relatively few EEG locations show statistically significant difference between the groups.

### EEG of Patients With CNP Reveals Frequency-Specific Temporal Signatures

**Figure 5:**
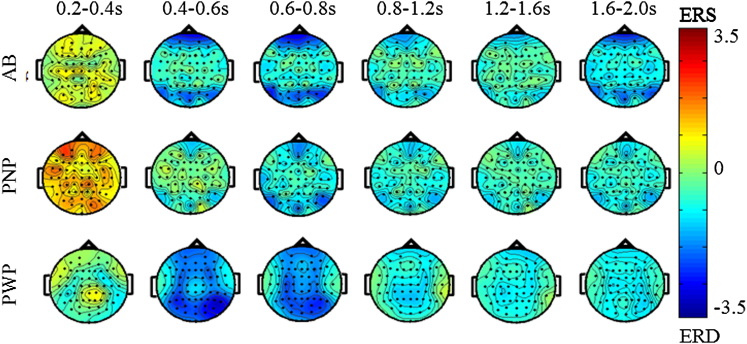
Scalp maps of ERS/ERS in the theta range (4–8 Hz) over different time windows for all 3 groups. The MI task was a repetitive tapping with both feet from t = 0 second until t = 3,000 ms.

**Figure 6:**
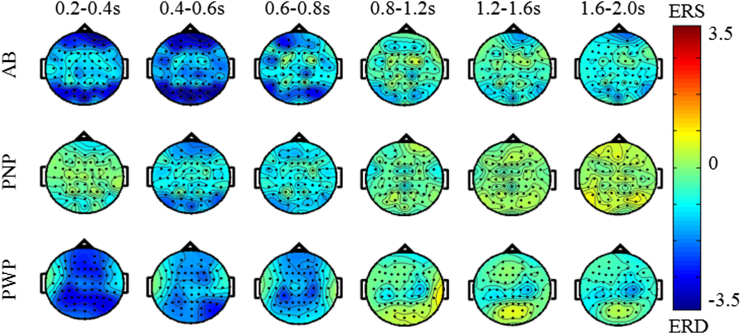
Scalp maps of ERS/ERS in the alpha range (8–12 Hz) over different time windows for all 3 groups. The MI task was a repetitive tapping with both feet from t = 0 second until t = 3,000 ms.

**Figure 7:**
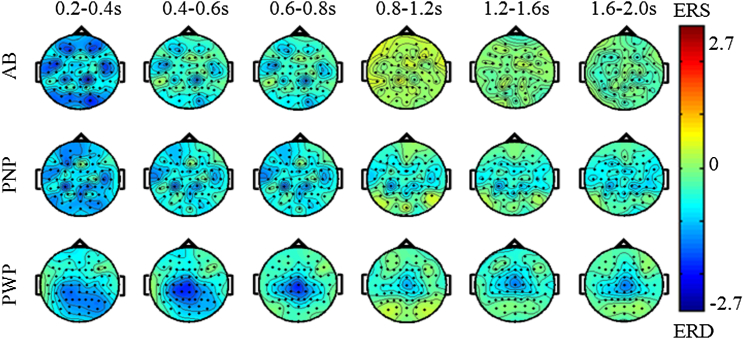
Scalp maps of ERS/ERS in the beta range (16–24 Hz) over different time windows for all 3 groups. The MI task was a repetitive tapping with both feet from t = 0 second until t = 3,000 ms.

MI induces dynamic cortical responses that cannot be captured using fRMI. In addition to showing that CNP causes a frequency specific activation pattern over several cortical areas, we show that this activation has a specific temporal pattern. As an example of this, a response to imagined movement of the feet is shown for all 3 groups for theta (Fig 5), alpha (Fig 6), and beta (Fig 7) band activities. Although participants repetitively imagined movement for 3 seconds, we show first 2 seconds only as this captures the important initiation of the task. In the theta band (Fig 5) and in the period 200 to 400 ms, all 3 groups exhibited ERS. Following this, in a period from 400 to 600 ms, the PWP group showed widespread ERD from the occipital to the frontal area until 800 ms. In the period 800 to 1200 ms, weak ERD was noted over the parietal (sensory) area. In the other 2 groups, theta ERD could not be noticed in the central area of the cortex. Thus, theta ERD over the sensorimotor cortex appears to be observed only in patients with CNP.

In the alpha band (8–12 Hz), Fig 6, the strongest ERD in the PWP group was in a period t = 200 ms to t = 800 ms predominantly located posteriorly, but for t > 800 ms, alpha ERD can be noticed at the lateral areas of the central region only. In the other 2 groups, distinctive ERD in the central area of the cortex could be noticed until t = 800 ms. In the AB group, ERD can also be noticed in the frontal and occipital area until the end of the analyzed period. The PNP group had weaker ERD in the frontal and occipital areas compared to the other 2 groups, and almost no visible ERD after t = 800 ms.

Finally, in the beta band (16–24 Hz) (Fig 7), all 3 groups had strongest ERD in the period t = 200 ms to t = 400 ms. In the period t = 200 ms to t = 600 ms, ERD in the PWP group can be noticed over the central, parietal, and occipital areas, but for t > 600 ms, ERD remained only at the central areas (Fig 7). ERD in the AB and PNP groups is widespread. No visible ERD can be noticed in the AB group after t = 800 ms, whereas some ERD over the central area could be noticed for both PNP and PWP groups until the end of the analyzed period.

## Discussion

This study provides evidence of altered spontaneous and evoked cortical activations in patients with CNP in the absence of peripheral nociceptive stimulation as a way to identify what aspect of the EEG might reflect a long-lasting alteration in brain behavior associated with chronic pain, thus further expanding on indicative results from fMRI.^17^, ^59^

In the relaxed EO state, PWP had higher alpha and theta power than the PNP group. The occipital alpha power in the EC state was comparable between PWP and PNP groups. PWP and AB groups showed similar EEG energy levels in both the EO and EC states. The PWP group also had significantly lower dominant frequency in the alpha band than both the AB and PNP groups. Increase in the theta power in presence of CNP is in accordance with previous studies,^11^, ^24^, ^36^, ^46^ whereas increase in the alpha power mirrors the observation of Sarnthein et al^46^ on a mixed patient group but disagrees with Jensen et al,^24^ who reported a decrease in alpha power. The PWP group had a reduced reactivity in the occipital alpha between the EO and EC states, which in this patient group has been attributed to a thalamo-cortical dysrhythmia.^11^ The low alpha power in the PNP group confirms results of previous studies on this patient group.^53^

During MI, the PNP group had weaker ERD than the AB group, whereas the PWP group had distinctively strong EEG signatures. The PWP group had significantly stronger alpha ERD over multiple electrode locations compared to both AB and PNP groups; this contrasts with group analysis of spontaneous alpha power in the EO state, where no difference was found between AB and PWP groups. Taken together, this indicates that strong ERD in the PWP group was not a simple consequence of a high alpha power in the reference period but can rather be attributed to the more intensive activation of the sensorimotor cortex. Of interest is that the PNP group had the weakest alpha ERD not only over central cortical areas but also over the frontal, parietal, and occipital areas, which are involved in higher-order cue-based movement planning. Beta band ERD in the PWP group also had a distinctive parietal location and was of stronger intensity than in the other 2 groups at equivalent sites. From all 3 groups, AB group had the shortest-lasting beta ERD.

A striking characteristic of the PWP group was a widespread ERD in the theta band, and as this is not an EEG frequency band commonly associated with movement or sensory-induced event-related spectral changes, it is likely to reflect the underlying CNP condition and may therefore be a putative signature of this disorder in both the relaxed^46^ and active states.

Although in this study we were not able to separate the influence of sensory loss and pain in the PWP group, EEG analysis between PNP and PWP groups showed statistically significant difference both in the relaxed state and during MI. This supports a novel theory of distinctive effect of sensory loss and of pain initiated by trauma leading to sensory loss.^32^

In this study we assessed cortical responses to motor tasks that associate with covert movement preparation and execution. We restricted our analysis to the surface cortical areas only, defined by a 10-10 system. Because of the nature of EEG measurement, we could only record the activity of surface cortical areas involved in processing of chronic pain, such as the sensory cortex and, to an extent, the frontal cortex. Although source localization techniques could be used to estimate ERD of deeper structures, such as the anterior cingulate cortex and insular cortex,^50^ such analyses were not performed in this study due to the need to have realistic head and brain model of the areas of interest. Surface cortical areas correspond to the areas that were typically targeted with noninvasive neuromodulatory treatments of pain.

The results of this study could be useful in informing neuromodulatory approaches for treatment of CNP. For example NF, tDCS, and rTMS^10^, ^12^, ^20^, ^22^, ^25^, ^26^, ^27^, ^28^, ^29^, ^30^, ^41^, ^45^, ^56^, ^48^, ^49^, ^51^, ^55^, ^56^ have all been considered as potential interventions to relieve CNP. Common to all these techniques is that they aim to modulate brain activity and typically target sites in the motor cortex, indirectly influencing cortical areas involved in a pain matrix.^21^ However, the choice of stimulation site and frequency of stimulus are still a matter of a debate.

NF treatment of chronic pain is typically based on increasing dominant activity (eg, alpha) and decreasing higher (eg, beta) frequency activity.^21^ It has been used in treatment of CNP,^25^, ^56^ complex regional pain syndrome,^22^ trigeminal neuralgia,^48^ migraine,^49^ and fibromyalgia.^27^ The mechanism of NF is not completely known, but it is believed that it facilitates global connectivity and after a prolonged practice induces neuroplasticity.^40^ The choice of rewarded or suppressed frequency bands and cortical location from which NF is provided are, however, often heuristically determined, based on a previous experience.^22^, ^25^, ^27^

In rTMS studies, cortical areas such as primary motor cortex,^20^, ^29^ primary sensory cortex,^26^ and parietal cortex^52^ have been targeted with pulse bursts in frequencies that varied from .2 to 20 Hz.^20^, ^29^ Through lateral cortical connections between the stimulated and other cortical sites, rTMS affects not only the stimulation site but also other cortical areas involved in the pain matrix, such as the prefrontal and sensory cortices,^29^, ^51^ possibly activating inhibitory circuits involved with pain reduction.^34^ It is believed that stimulation with frequencies in a range 10 to 20 Hz, which has the largest effect on reducing pain, restores the intracortical inhibition and causes increase in EEG power.^29^

For pain treatment, anodal tDCS over electrode location C3 or C4 (primary motor cortex) was proposed.^10^, ^12^, ^42^ Anodal tDCS increases excitability, whereas cathodic tDCS decreases it, analogous to mechanisms supporting long-term potentiation and depression, respectively.^35^

Contrary to neuromodulatory treatments of pain, the MI task induces desynchronization, that is, reduced EEG power and increased cortical activation of the sensorimotor cortex. We hypothesize that the areas of largest ERD, that is, most active during MI, might be the most responsive to neuromodulatory treatments. Due to the nature of EEG recording we could not be certain of the contribution of different cortical areas to recorded ERD. However, theta, alpha, and beta ERD showed a distinctive spatial distribution, which indicates that for different stimulation frequencies of rTMS or NF, there might be distinctive optimal cortical areas. Further advanced source analysis methods, however, would be required to confirm that areas of strongest theta, alpha, and beta activity really have spatially different location of their sources. Finally, our results indicate that in paralyzed patients, due to a posterior shift of the strongest ERD, which might be related to functional and anatomic changes of the motor cortex,^60^ the location of most reactive cortical areas might not be the same as in the other patient groups suffering from CNP.

A noteworthy finding of this study is that increased ERD over the sensorimotor cortex in the PWP group is widespread, indicating a possibility that MI in the presence of CNP equally affects the cortical presentation of painful and nonpainful limbs. This widespread effect indirectly supports the results of neuromodulation/neurostimulation studies, which showed that although it is important to modulate activity of the motor cortex, it is not necessary to target cortical areas corresponding to the painful part of the body.^25^, ^28^, ^29^, ^56^

It should be mentioned that although this EEG study demonstrated distinctive brain activity in patients with CNP, it could not confirm any related anatomic or functional changes. Therefore, it is possible that different factors such as medication, disuse reorganization, anxiety, or depression influenced brain activity recorded by EEG. A disuse reorganization unrelated to CNP should be present in both PWP with PNP groups, but these 2 groups had distinctive EEG responses both in a relaxed state and during MI. Anxiety and depression might have contributed to EEG signatures of the PWP group,^42^ though these patients showed no statistically significant difference in EEG power in a relaxed state compared to the AB group. Antiepileptic drugs and antidepressants that were used by patients for treatment of CNP might have affected their relaxed-state EEG, in particular in the theta band.^5^, ^58^ Antidepressants increase EEG amplitude in the theta and the higher beta (>20 Hz),^58^ whereas antiepileptic drugs are known to slow down the dominant frequency and increase the energy in the theta and delta bands.^5^ Antispastic drugs taken by 2 patients targeted gamma-aminobutyric acid receptors and could potentially also increase the energy level in the theta and delta bands.^54^ However, evidence from the literature shows that theta band power is reduced in patients undergoing surgery for CNP,^46^ suggesting that increased power in the theta band is most likely related to pain, not to medications alone. Ideally the study should include another group with CNP without other neurologic condition affecting EEG, to assess the separate effect of pain.

In summary, our study showed that CNP is associated with changes in spontaneous and evoked EEG. As a result, SCI patients with and without CNP show significantly different signatures of spontaneous and evoked EEG. In the relaxed state, CNP is characterized by the increased power in the theta and alpha bands and shift of the dominant alpha frequency toward lower values. During MI, CNP is characterized by a dynamic, frequency-dependent increase of ERD over the sensorimotor and parietal cortices, not somatotopically restricted to painful parts of the body. Results of this study may aid in the design of neuromodulation-based therapies.
